# Stakeholder perspectives and experiences of the implementation of remote mental health consultations during the COVID-19 pandemic: a qualitative study

**DOI:** 10.1186/s12913-023-09529-x

**Published:** 2023-06-13

**Authors:** Emer Galvin, Shane Desselle, Blánaid Gavin, Etain Quigley, Mark Flear, Ken Kilbride, Fiona McNicholas, Shane Cullinan, John Hayden

**Affiliations:** 1 School of Pharmacy and Biomolecular Sciences, Royal College of Surgeons in Ireland, Dublin, Ireland; 2 Touro University California, California, CA USA; 3 School of Medicine, University College Dublin, Dublin, Ireland; 4 National University of Ireland, Maynooth, Ireland; 5 Queen’s University Belfast, Belfast, UK; 6 ADHD Ireland, Dublin, Ireland; 7 Children’s Health Ireland, Crumlin, Dublin, Ireland; 8 Lucena Child and Adolescent Mental Health Service (CAMHS), Rathgar, Dublin, Ireland

**Keywords:** Telemedicine, Telemental health, Telepsychiatry, COVID-19, Implementation, Remote consultations

## Abstract

**Background:**

Remote mental health consultations were swiftly implemented across mental health services during the COVID-19 pandemic. Research has begun to inform future design and delivery of telemental health services. Exploring the in-depth experiences of those involved is important to understand the complex, multi-level factors that influence the implementation of remote mental health consultations. The aim of this study was to explore stakeholder perspectives and experiences of the implementation of remote mental health consultations during the COVID-19 pandemic in Ireland.

**Methods:**

A qualitative study was conducted whereby semi-structured, individual interviews were undertaken with mental health providers, service users, and managers (*n* = 19) to acquire rich information. Interviews were conducted between November 2021 and July 2022. The interview guide was informed by the Consolidated Framework for Implementation Research (CFIR). Data were analysed thematically using a deductive and inductive approach.

**Results:**

Six themes were identified. The advantages of remote mental health consultations were described, including convenience and increased accessibility to care. Providers and managers described varying levels of success with implementation, citing complexity and incompatibility with existing workflows as barriers to adoption. Providers’ access to resources, guidance, and training were notable facilitators. Participants perceived remote mental health consultations to be satisfactory but not equivalent to in-person care in terms of quality. Views about the inferior quality of remote consultations stemmed from beliefs about the inhibited therapeutic relationship and a possible reduction in effectiveness compared to in-person care. Whilst a return to in-person services was mostly preferred, participants acknowledged a potential adjunct role for remote consultations in certain circumstances.

**Conclusions:**

Remote mental health consultations were welcomed as a means to continue care during the COVID-19 pandemic. Their swift and necessary adoption placed pressure on providers and organisations to adapt quickly, navigating challenges and adjusting to a new way of working. This implementation created changes to workflows and dynamics that disrupted the traditional method of mental health care delivery. Further consideration of the importance of the therapeutic relationship and fostering positive provider beliefs and feelings of competence are needed to ensure satisfactory and effective implementation of remote mental health consultations going forward.

**Supplementary Information:**

The online version contains supplementary material available at 10.1186/s12913-023-09529-x.

## Background

The COVID-19 pandemic and its associated lockdowns necessitated the virtual delivery of mental healthcare to reduce spread of the virus while maintaining continuity of care. Prior to the pandemic, systematic reviews reported that “telemental health”, the use of telemedicine to provide mental health care, is equally effective and acceptable as in-person mental health care [1–3]. Despite this evidence, the widespread implementation of telemental health had been slow up to this point [4]. This research-implementation gap existed because of a number of regulatory, technological, and clinician barriers [5]. However, the swift adoption of telemental health during the pandemic has highlighted various implementation successes and challenges.

Accompanying the rapid rise in the adoption and use of telemental health is a body of literature reporting on this recent transition to remote care [6–9]. Many patients and clinicians reported being satisfied with telemental health during the pandemic [10, 11], with benefits of flexibility and convenience being cited in the recent literature [12]. Moreover, studies have shown that patients and clinicians are open to using telemedicine in mental health care beyond the pandemic [6, 13]. However, challenges such as building rapport, lack of non-verbal cues, and reduced confidence in diagnosing patients remain as concerns [14]. Many studies of this recent telemental health implementation have been conducted in the USA [14], so there is a need for rigorous, high-quality research in other countries with different mental health care contexts. The focus of this study are remote mental health consultations. These are phone and video consultations between mental health providers and service users that swiftly replaced in-person consultations during the COVID-19 pandemic.

The reported benefits of telemental health, combined with satisfaction by patients and providers, suggests that it will continue to play a role in mental health care [15]. The extent of this role is less clear, with an awareness that telemental health may not be suitable for all individuals, circumstances, and contexts [16]. To ensure its appropriate and effective use, the barriers and facilitators to this recent adoption warrant investigation. Exploring the views of patients and providers is of great importance when a novel modality is being introduced into a health service, to understand the implementation challenges and successes from diverse standpoints [17, 18].

Within the context of the COVID-19 pandemic, many quantitative studies have investigated satisfaction and uptake of telemental health by providers and patients [12, 19]. A recent systematic review by the authors found that quantitative studies of patient and provider perspectives regarding telemental health implementation were of low and moderate methodological quality [14]. Further, these studies provided a surface-level investigation of barriers and facilitators to telemental health implementation. There is now a need for qualitative studies with service users and staff to gain in-depth insights into the factors that may ensure, and even optimise, engagement with remote mental health care [13, 20]. Employing a qualitative approach is essential to elucidate the reported challenges and successes in the recent literature, including perceptions of the therapeutic relationship and provider satisfaction [21]. Indeed, qualitative studies have begun to explore the experiences of service users and providers with telemental health during the pandemic [22, 23], though less research has explicitly focused on implementation [14]. Many of these studies have focused on experiences within specific contexts, namely hospital outpatient settings, and on homogenous patient and provider groups [14]. An important facet on qualitative research in implementation science is a focus on multi-level stakeholders, to gain a comprehensive understanding of barriers and facilitators to implementation of an innovation [24].

The aim of this study is to explore the perspectives and experiences of stakeholders of the implementation of remote mental health consultations during the COVID-19 pandemic.

## Methods

### Design and setting

The study employed a qualitative design that enabled the exploration of the views and experiences of various stakeholders of the implementation of remote mental health consultations. Data collection and analysis were conducted using an essentialist approach. According to an essentialist approach, participants’ responses are understood to express their intended meaning and reflect their own thoughts and experiences [25]. This was achieved by focusing on the “reality” of the participants’ experiences and generating themes at the semantic level [25]. The setting for this study was mental health services in Ireland, including primary care, outpatient services, community-based services, private services, and counselling services.

### Conceptual framework

The Consolidated Framework for Implementation Research (CFIR) was employed to inform this qualitative study [26]. This framework categorises implementation determinants into five domains [26]. These domains are intervention characteristics, outer setting, inner setting, characteristics of individuals, and process. This framework was chosen for this study as it acts as a guide for systematically assessing the implementation of an innovation [26]. The CFIR has been commonly employed in post-hoc analyses of what facilitated or hindered implementation of an innovation [27] and has been previously applied in the context of telemedicine implementation [28]. While traditionally used to evaluate the implementation of a well-planned, evidence-based intervention, the novel approach of the CFIR in this study allowed for the evaluation of a swiftly-implemented innovation. In this study, the CFIR informed the topics to be explored in the interview guides. The CFIR was also employed *apriori* at the data analysis stage.

### Participant recruitment and sampling

We employed maximum variation purposive sampling to obtain a wide range of views and perspectives from key stakeholders, including service users, mental health providers, and managers. The use of maximum variation sampling in this study allowed for the exploration of the “common and unique manifestations” of the target phenomenon (remote mental health consultations) across a range of demographically-varied participants [29]. In implementation science, “key stakeholders” are conceptualised as individuals who play a role or are impacted by the implementation of an innovation [24].

Our recruitment strategy aimed to recruit participants from three groups; service users, mental health providers, and managers/implementation leaders. The following eligibility criteria was applied. Firstly, the mental health provider group included health care professionals that had undertaken at least two remote mental health consultations with service users in the past year. Secondly, the service users included adults aged over 18 who had partaken in at least one remote mental health consultation with a health care provider in the past year. Parents and caregivers were also eligible to take part. Finally, managers and implementation leaders from organisations and voluntary, or professional, bodies involved in implementing remote consultations in the past year were recruited. Participants were recruited through personal and professional contacts. The study was advertised to mental health providers at a university webinar. Advertisements were also placed on the lead researcher’s social media page, and participants were asked to contact the lead researcher via email.

The sample size was informed by previous studies and the concept of information power [30], guided by Sim and colleagues’ [31] considerations for determining qualitative sample size *apriori*. Based on similar studies [8, 32], it was anticipated that an initial sample of 15 participants would be recruited, with a minimum of five interviews per participant group. The informational power of the interviews was also considered. Information power in sampling specifies that the more information a sample holds in relation to the study aims, the less number of participants are needed [30]. Information power was informed by a number of considerations, including the quality of dialogue (due to the interviewer’s experience) and the broadness of the aim (due to the heterogeneity of the participant groups) [30]. Data collection and analysis were an iterative process, with sampling continuously assessed based on the informational power of the interviews.

### Data collection

Semi-structured interviews were conducted by the lead researcher (EG) between November 2021 and July 2022. A total of 19 interviews were conducted over video call, using Zoom and Microsoft Teams software. EG is a female, PhD researcher trained in qualitative methodology and interviewing techniques. EG did not have experience engaging in remote mental health consultations as a service user, so had little practical knowledge of what they entailed. Participants were informed that the study comprised part of the lead researcher’s PhD project. Two mental health providers were known to the researcher through professional networks.

A semi-structured approach was used. This ensured that core questions were asked in every interview with the flexibility to augment with more probing questions when appropriate. The interviews were recorded using an audio-recording device and lasted an average of 30 minutes in duration (ranging from 16 to 52 minutes). Field notes were documented to ensure reflective practice, and to inform data analysis and interview guide refinement.

### Interview guides

The interview guides were informed by the CFIR, a literature review, and input from an expert advisory group (See Additional file 1 for the mental health provider interview guide). Example questions for the mental health provider group included “*Compared to in-person consultations, what do you think are the advantages of remote consultations?*” (Intervention characteristics: relative advantage) and *“What changes would you make to remote consultations to make them fit or work more effectively in your setting?”* (Intervention characteristics: adaptability). Three different interview guides were created with a similar line of questioning for service users, mental health providers, and managers/other stakeholders. Topics of discussion included technological issues, advantages and disadvantages, and lessons learned. The interview guide was then piloted with a psychiatrist. Modifications to the interview guide were made to improve question flow and clarity, and to include additional questions. Each guide was iteratively refined during the study when appropriate. For example, in the interview guide used with service users, a question was added to ask about methods used to enhance privacy when participating in remote consultations.

### Data analysis

All transcripts were entered into QSR NVivo 12 to facilitate data analysis. The lead researcher transcribed all interviews verbatim. Thematic analysis was conducted by the lead researcher using an inductive and deductive approach [25]. As described below, the six steps of thematic analysis were followed, addressing Lincoln and Guba’s [33] criteria for trustworthiness throughout the analysis [34]. The first step involved familiarisation with the data through listening to the audio-recordings and reading the transcripts and field notes. The lead researcher documented their thoughts about potential codes and themes at this stage. The second step involved conducting line-by-line coding on the transcripts using inductive coding. Memos were created at this stage to capture interesting ideas in the interviews. Once all the data were coded and collated, the CFIR was utilised to develop broad, overarching codes to organise the data (Step 3). These deductive codes then formed main themes. An adapted CFIR codebook was employed at this stage to ensure consistency with coding across transcripts. Subthemes were developed inductively and deductively using relevant sub-constructs of the CFIR. The lead researcher kept detailed notes of the development of themes and subthemes to help establish confirmability. Miscellaneous codes that did not fit within the codebook were kept in separate nodes in NVivo to ensure that they were not lost. The fourth step involved reviewing the themes and subthemes. At this stage, the lead researcher reviewed the coded data extracts of each theme and subtheme to ensure that they were coherent with each other. The participants’ raw data was also reviewed to ensure that the themes accurately reflected the participants’ words. The fifth step involved clearly defining and naming the themes. A brief description of each theme was created. The sixth, and final step, involved the write-up of the results.

### Patient and public involvement

A representative from the Patient and Public Involvement (PPI) partner, ADHD Ireland, contributed to the study design and the conception of the wider project. The PPI representative contributed to the development of the interview guide and protocol development. They were also consulted in the reporting of the findings. The representative met with the research team via Zoom meetings and also communicated feedback via email. The representative was not compensated for their contribution, but was included in the study authorship. Input for the interview guide was also given by medico-legal experts and attendees at a health law and ethics forum. An expert advisory group, comprising of psychiatrists and implementation science experts, contributed to the design of the study and interview.

### Ethics

Ethical approval was obtained by the Royal College of Surgeons in Ireland (RCSI) Research Ethics Committee (Reference number: 202105014). Written consent was obtained by all participants. Before the interviews, the interviewer explained the aim of the study and participants were reminded that their participation was voluntary and that they could withdraw at any time. Participants were also assured that their confidentiality would be protected and that any identifying details would be pseudonymised.

### Rigour and trustworthiness

To ensure rigour, the study was reported in accordance to the consolidated criteria for reporting qualitative research (COREQ) checklist [35] (See Additional file 2). To achieve dependability and confirmability, clear and detailed descriptions of the research processes were provided. In addition, the lead researcher (EG) kept detailed records of decisions made during the analysis stage [36], including thoughts about codes and themes. A coding framework for the deductive analysis was developed *apriori*, and was adapted and revised by members of the research team. Whilst the coding was led by the lead researcher, the research team was debriefed at the various stages and provided input on the final themes and subthemes. Finally, the lead researcher (EG) kept a reflexivity journal to gain and maintain self-awareness of their perspectives and their possible effect on the interpretation of findings [36].

## Results

**Fig. 1:**
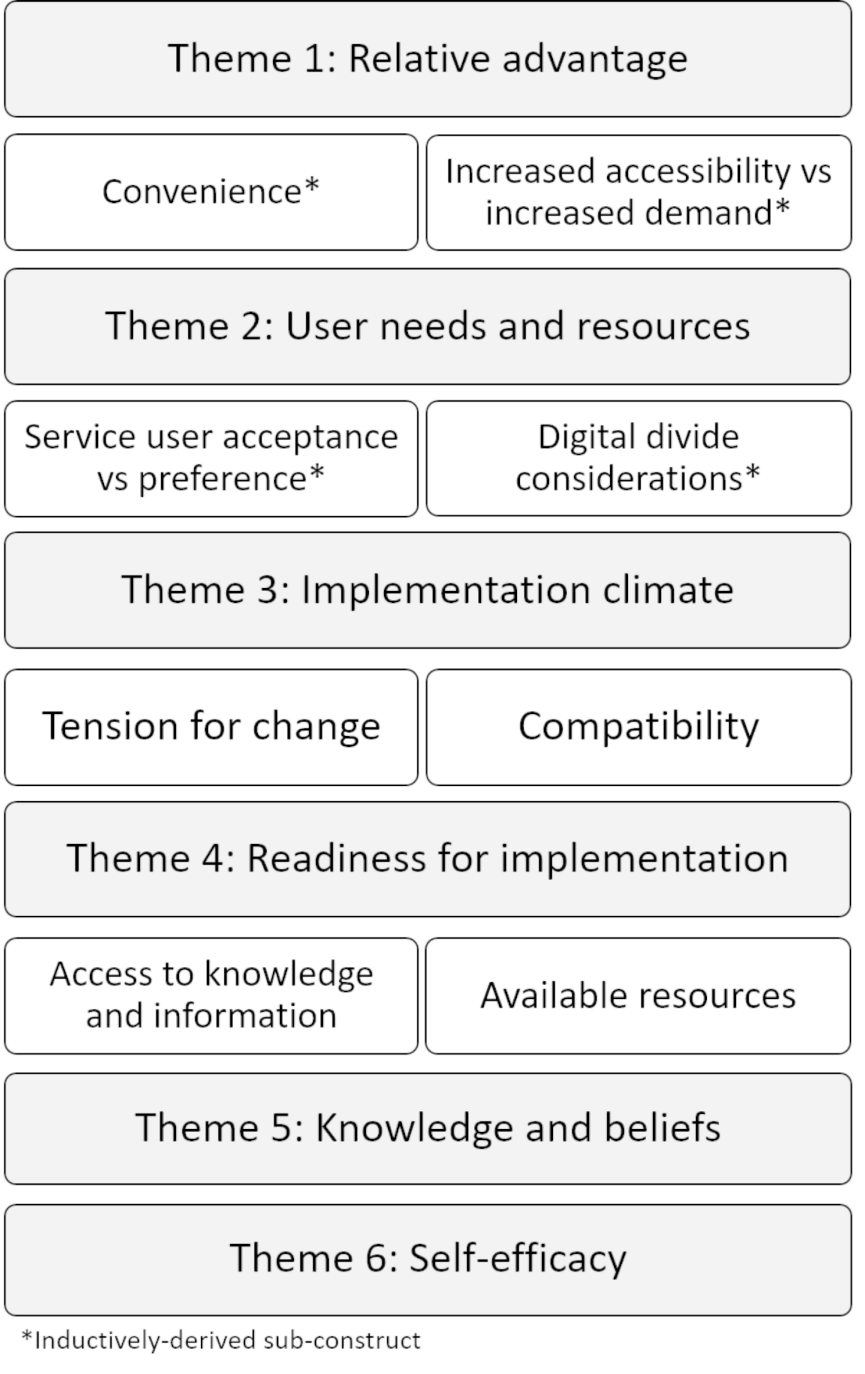
Themes and subthemes

Nineteen stakeholders participated in interviews, comprising of mental health providers (*n* = 9), service users (*n* = 5), service managers (*n* = 4), and a manager from a professional body (*n* = 1). The mental health providers comprised of psychologists and trainee psychologists (*n* = 3), general practitioners (*n* = 2), psychiatrists (*n* = 2), a pharmacist (*n* = 1), and a psychotherapist (*n* = 1). The full demographic characteristics of the sample can be seen in Additional file 3. The aim of this study was to explore the perspectives and experiences of stakeholders of the implementation of remote mental health consultations during the COVID-19 pandemic. The data analysis revealed themes and subthemes relating to these views and experiences (See Fig. 1). These themes relate to the most relevant constructs of the Consolidated Framework for Implementation Research (CFIR).

### Relative advantage

In this theme, participants discussed the relative advantages of remote mental health consultations compared to in-person consultations in relation to their convenience and access.

#### Convenience

Participants recognised the convenience of remote consultations for service users as an overwhelming advantage. Service users appreciated being able to partake in remote consultations from anywhere, not having to travel, or take additional time off work. The cost-savings in comparison to travelling to an in-person appointment were discussed by providers working with refugees on low incomes and by providers working with users in rural areas, where a long car journey would be expensive. Importantly, in the context of the pandemic, being able to attend appointments while ill or isolating with COVID-19 was acknowledged as an advantage. Providers recognised that for parents with children, the removal of a long car journey was a benefit, resulting in less missed school and less disruption to the child’s routine:

“But what I find is that it’s worked so well and you know, across the board patients are saying to me like, you know, this is so much handier for me like I have kids now where I see them and their parents are going collecting them from school, they’re in their car, they’re coming from their training. It’s a much less of a burden on their lives.”

(Child and adolescent psychiatrist, Provider 9)

Whilst many service-users reported a preference to be in-person, the inherent convenience of remote consultations was a notable advantage:

“Like, you know, I’d say a lot of people just feel a lot more comfortable doing the consultation over the Zoom because they don’t have to prepare, get bus fare together or run out and catch the bus on time or if it’s rainy outside, they don’t have to battle the rain, or the elements, or even if they feel a bit sick with the flu, it’s all on Zoom.”

(Service user 5)

This “trade-off” between convenience of remote consultations and service-user preference was acknowledged by a manager of a counselling organisation:

“Obviously they don’t prefer it [in-person] enough to get in here, but they prefer to be in-person.”

(Manager 3)

#### Increased accessibility vs. increased demand

The increase in accessibility to care was discussed as an important advantage of remote consultations. Providers acknowledged that remote consultations made it easier for people who may have previously struggled to attend in-person to now attend, such as those with anxiety, those who are neuro-divergent, and those with depression:

“In our group of patients, it has the advantage in that if someone is very unwell, or has high anxiety levels, or has depression, they may lack the motivation to actually come into the hospital and attend an outpatient consultation. So for those groups of patients, they don’t have to leave the house, they can do it from home and still get access to that kind of high quality service.”

(Pharmacist, Provider 2)

With the move to remote services, managers and providers, primarily working in private mental health services, expressed surprise at the increased reach of their services, no longer being restricted by geographical distance. As manager of a low-cost counselling organisation working with a marginalised group described:

“I was delighted that we were able to move and do that and facilitate and not only facilitate the existing client group but actually open it up to many others.”

(Manager 2)

However, with this increased reach, this manager described experiencing an increased demand on their services and trying to manage this increased demand with relatively limited resources. The challenge of attempting to return to an in-person service was also described, given that the service had grown dramatically during the pandemic:

“And more people are coming in, but again, that presents more challenges because then it’s how do you manage that? How do you sustain that work? […] And if you go back face-to-face, how do you kind of transition back into in-person sessions? […] You only have a small team of counsellors. You know, that’s the challenge.”

(Manager 2)

### User needs and resources

Participants described the extent to which the preferences, needs, and resources of service users were considered and prioritised, in addition to the needs and resources as described by service users themselves.

#### Service user acceptance vs. preference

Providers and managers described some initial reluctance among service users towards remote consultations at the start of the pandemic, describing that some users did not feel comfortable with video consultations. Providers described that despite this initial reluctance, service users were generally accepting of remote consultations and appreciated being able to continue to access care. Within the interviews, there was a sense that service users were willing to make the most of remote consultations and that they could meet their needs for the time being:

“So it was a good kind of holding piece. You know, it held me where I was and kind of was able to help me with the problems I was having at the time.”

(Service user 4)

Providers observed that the level of desperation of service users played into their decision to partake in remote care, particularly for parents who were waiting for assessments for their children, further highlighting the demand for services at this time. Most service users described not having any experience of in-person mental health consultations so did not have any expectations about what remote consultations would entail. Providers and managers expressed that service users were keen to return to in-person services, with service users themselves expressing a preference for in-person services care in the interviews.

#### Digital divide considerations

Providers and managers acknowledged that one of the main challenges was the presence of “digital divide” barriers, whereby service users did not have access to adequate internet connection and resources (i.e. devices, data plans, email addresses) to partake in video consultations:

“Some people didn’t really have the facility to do online […] other people then they just, they weren’t, they were like I don’t know how to use that, I don’t have access to a laptop, so can we just do phone? […] just I suppose even availability of the whatever you need, like the phones were definitely not great, you know you really kind of need at least a tablet, if not a laptop.”

(Trainee clinical psychologist, Provider 8)

For providers working with refugees and service users in rural areas, these barriers were particularly prominent. As highlighted above, the cost-related travel savings were of particular advantage to these service users, but their engagement was hindered by the presence of digital barriers.

These digital divide barriers also pertained to service users’ digital literacy. Whilst the service users interviewed in this study expressed that they were quite competent with remote consultations, providers acknowledged the low digital literacy of some of their service users, specifically those from marginalised groups. Managers and providers described considering the needs of their users when choosing which platforms to use for video consultations, for example choosing a simple and easy-to-use platform that did not require service users to have an email address. The novelty of these new set of issues to consider was expressed by providers, including by a psychologist working with refugees:

“Having to have conversations with clients about what kind of devices they have or what kind of data they have, you know, like obviously we’ve never had to ask questions like that before.”

(Psychologist, Provider 3)

### Implementation climate

This theme described participants’ perceptions of the perceived need for remote consultations (*Tension for change*) and how well remote consultations fit with existing workflows and systems (*Compatibility*).

#### Tension for change

The rapid implementation of remote consultations occurred in response to the need to stop the spread of the COVID-19 virus. Providers and managers described a swift and sudden transition to this remote way of working, driven by this urgent need:

“A lot of people were concerned about the continuity of service and you know, obviously their established relationships with clients. So we needed to introduce some options.”

(Manager 5)

There was a sense of participants, providers and service users, not having a say in this move to remote working, having to go along with this novel modality. Uncertainty about when in-person care would resume contributed to some of the decision-making of service users towards accessing remote care:

“I suppose the main benefit was, it was either do online therapy or wait until when we could do it in-person and we didn’t really know, there wasn’t much guidance at the time when that was going to happen.”

(Service user 4)

For the most part, in-person consultations were cancelled, and providers and organisations moved quickly, in some cases in the space of a couple of days, to get remote consultations up and running. There was a “just got on with it” approach with little time for planning:

“Well, uhm, I guess it was a question of needs must.”

(Psychiatrist, Provider 4)

Ehm, I think we were all a bit like rabbits in the headlights

(GP, Provider 5)

Providers and managers acknowledged that the pandemic disrupted the traditional way in which healthcare was delivered, prompting organisations and providers to be flexible and provide options to their service users.

#### Compatibility

Providers and managers described the compatibility of remote consultations with existing workflows, processes, and systems. For some organisations, the complexities involved with trying to set-up video conferencing, as discussed below, resulted with video consultations not being implemented at all. Providers, namely those in general practice, described various reasons for failing to implement video consultations, such as service user difficulties with technology, not being able to embed video platforms in the practice software, service user discomfort and low provider motivation in a highly stressful context:

“We dabbled with video. Didn’t really find it helped that much, so that didn’t last long […] a combination of things probably. A little bit of technology. A little bit about, ehm timing, you know […] with video it was rather dependent on everybody being where they should be at the right moment in time, with cameras working and everything. And then even at that you know, I found a lot of people were not necessarily overly comfortable with it and it was just, it was just a lot less- It was a lot more hassle by and large […] You know, part of that is ourselves. If we had a more structured setting in the practice it might have worked. But COVID was kind of chaotic anyway.”

(GP, Provider 5)

Here, the interplay of various factors resulted in video consultations not working out as planned. Instead, phone consultations were used as the primary method to provide remote care. For one provider who worked primarily with non-English speaking refugees, trying to integrate an interpreter into the remote consultation was an added level of complexity and posed its unique challenges to the quality of care and therapeutic relationship.

General practitioners (GPs), who worked mainly over the phone, noted that phone consultations were not valued the same as in-person consults by administrative staff, whereby multiple phone consultations could be scheduled into one appointment slot. One GP described the advantage of now having protected time to conduct phone consultations, compared to pre-pandemic:

“Whereas now we within our practice software, there’s actually, we actually have an option for a phone consult. So that means that you’ve got protected time to do it, whereas before you’d have given yourself a task, follow this person up in two weeks, give them a call. Like say if it was a mental health problem and you want to see if someone was responding, but that was extra work. So it was kind of more part of your admin than that than that would have been a routine call. So in some ways it’s better now that we have a more structured approach to it.”

(GP, Provider 6)

Another change that was experienced by providers was an increase in their workload. Providers discussed the additional workload they did preparing service users for the virtual visits, including informing them on how to set up the screen and their environment. A psychiatrist working privately explained that she had to take on additional staff to deal with the increase in administrative workload relating to the implementation of remote consultations.

### Readiness for implementation

This theme described the providers and organisations readiness to implement remote consultations, including the ease of access to knowledge and information about them (*Access to knowledge and information*) and the level of available resources needed for their implementation and ongoing use (*Available resources*).

#### Access to knowledge and information

Providers and managers discussed the availability of training and guidance as a facilitating factor. In addition to participating in training on how to use platforms, providers described participating in training on how to conduct therapeutic work online. Providers also described receiving training and guidance from their professional bodies, with a manager of a professional body detailing how they were able to quickly provide necessary guidance and answer questions from their members at the onset of the pandemic:

“There were a few questions around […] you know, how to work online safely and which platform to use. We try not to be this prescriptive because it’s very difficult, but also we were just looking at those areas what the platform should provide to be considered safe, safe as well. They were- I’m just looking at the questions there. Also online supervision. Ehm, insurance for online work.”

(Manager 4)

Managers described providing training to their staff, with a manager of a counselling organisation detailing going a step further by creating an autonomous training platform for their therapists. Providers and managers talked about getting advice from experts on how to implement remote consultations and work effectively online. Managers greatly appreciated having access to experts for advice and very much valued their opinions:

“And we were very lucky to have somebody who did some training with us in both of them [sic] areas. He’s an experienced, long term psychotherapist who’s been providing training in online therapy for a good few years and also has worked in the IT sector. So you know the combination was perfect. He came in and did some- He did a number of training sessions with the team.”

(Manager 2)

At the start of the pandemic, participants reported that it was challenging to keep up with the quickly-changing, and sometimes conflicting, guidance, from various bodies and organisations. In particular, changing guidance about safety of video platforms created confusion and concern among providers and managers, with a therapist explaining that they stopped using a particular platform when there was scepticism about its end-to-end encryption:

“I would- I was sceptical at some points around safety, you know, and I wasn’t sure about the assurances in the GDPR and so on and when they were doubts, then I tried to switch off it again when possible, you know, just until I was sure. So that’s kind of disconcerting.”

(Psychotherapist, Provider 1)

A manager described that their organisation created their own video platform in response to confusion over the data security of commercial software. With time and experience, these concerns lessened and were replaced with increased trust in the platforms.

#### Available resources

Providers and managers described the resources needed to implement remote consultations. Managers discussed investing a considerable amount of time, money, and effort in getting a remote service off the ground. In contrast, one manager described having the infrastructure to do remote consultations already in place, as the organisation was planning on offering online services at some point:

“So we have our own remote platform that conducts video sessions from so everything is in-house. We don’t use third party software. […] Because we had that infrastructure in place already, it meant that we could comfortably operate.”

(Manager 1)

Providers acknowledged that video platforms were easily accessible and easy to use, as did service users:

“It’s fairly easy to use. Like at the time like I would have been using it a good bit for work. So I was like totally used to how it worked and pure lucky, my connection was grand the whole time ‘cause sometimes it would play up a bit. So yeah, no, I thought it was really easy to use.”

(Service user 2)

For the most part, providers described having sufficient equipment to get started with remote consultations. However, for a minority of providers, the associated move to remote working raised new considerations about the resources needed for providers to conduct consultations from their home setting. One psychiatrist described adjustments they made to be able to work effectively from their home:

“I mean I had to upgrade my broadband. I had to, you know, get cameras and headphones and all of that. I had to modify the workspace at home to try and make that work, had to work from home, which was a big uh, a big change.”

(Psychiatrist, Provider 4)

### Knowledge and beliefs

This theme described participants’ knowledge and beliefs about remote mental health consultations, and how these views related to implementation. A common narrative across all interviews was the perceived importance of in-person consultations, which seemed to influence participants’ perceptions of the quality of remote care, both from a relationship-building, and an effectiveness, standpoint. Providers, in particular, expressed a belief that therapy was more suited to be in-person, discussing the difficulties establishing a therapeutic relationship, the lack of non-verbal cues, and challenges with specific types of therapy (e.g. exposure therapy) as issues.

Another reported challenge was that of creating a therapeutic space online. A manager, who was also a trained psychotherapist, acknowledged the importance of creating a contained space where service users felt safe and stressed the importance of this containment for service user engagement in therapy. The importance of participating in a contained space was emphasised in the service user interviews. A service user described that with remote therapy, she is left with her trauma in her personal space after her session, preventing her for going into sensitive topics:

“Usually I was doing it from a bedroom […] And you don’t necessarily want to bring up something that is difficult for you to talk about it or is traumatic because then you’re going to be left in your personal space, you know you’re gonna log off Zoom and you’re there. Whereas when you go to therapy in another room, you talk about your problems there and you leave it there, and it’s contained in that space.”

(Service user 4)

Fear of being overheard by household members was also discussed by service users and given as a reason for not divulging sensitive issues. In addition, it was noted by one service user that the online format acted as a “barrier” to expressing her feelings:

“Whereas online, I don’t know, there’s a bit of, a small bit of a barrier or something like it could be easier to maybe hide how I’m feeling or something like that. […] whereas I feel like online. It’s a bit easier to maybe have a facade or not, kind of, let out, like if your problem is maybe not expressing emotions, it’s probably easier to continue to do that.”

(Service user 1)

These beliefs about the quality of remote care appeared to play into participants’ preference for in-person services, with many participants reporting a return to in-person consultations following the pandemic. The belief that that remote consultations were of lesser “value” than in-person consultations was present in the service users’ discourse:

“There was a much more diminish on returns on them [remote consultations] than going to see someone in-person.”

(Service user 3)

Despite this preference, providers acknowledged appointment types that can be effectively done remotely, such as follow-up appointments, transactional appointments e.g. prescriptions, and some types of assessments and talk therapies with established users. Furthermore, providers emphasised the utility of remote consultations in situations when it was preferable and more convenient for the user, emphasising the importance of user’ needs and preferences.

### Self-efficacy

Mental health providers described their beliefs in their own capabilities to conduct remote mental health consultations. Pre-pandemic, providers reported having little experience with using remote consultations. This was usually in exceptional circumstances, such as when a service user was located some distance away. Despite this lack of prior experience, some providers reported feeling comfortable and confident with conducting remote consultations. Initially, providers questioned their ability of how to deal with challenges such as managing service user distress, service user risk, and suicidal ideation online:

“So yeah, so that would have been my only concern. Am I able to manage this person’s distress? Can I help them feel safe and supported if they become highly distressed? You know, for example, if anyone expresses any suicidal ideations, they could be 50 miles away from me, you know, what would I do?”

(Psychologist, Provider 3)

Providers described strategies to mitigate any safety risks to the service user, including obtaining a back-up phone number, next-of-kin details, and local emergency contact details. There was a sense of a loss of control of the providers being able to manage situations that they would usually be able to manage in-person, such as not being able to control who was in the service user’s home environment. This was of particular concern to providers working with adolescents, who expressed unease at the possibility of parents being in the background without their knowledge, creating challenges around confidentiality.

Providers also discussed finding it challenging to manage “inappropriate” situations, such as when children and families partook in consultations from their bed or wearing pyjamas to the consultations. This increase in informal behaviours signalled a change in dynamic from the traditional service user-provider relationship:

“Some people were there, essentially in their pyjamas or with their hair wet, or in a way that like you wouldn’t just see someone rolling up here in outpatient appointments normally and maybe the kids are sitting there in their PJs and, just again, it just had an influence on the dynamic.”

(Child and adolescent psychiatrist, Provider 9)

The disruption of this dynamic displayed a change in the level of perceived seriousness of the consultation and signalled to this psychiatrist that these service users had a lack of “respect” for the process. To another provider, this increase in informality, specifically on phone consultations, demonstrated service user comfort and relaxation in their home environment, which they viewed as a positive:

“Maybe they’re able to walk around their house, maybe they’re able to just like kind of slump in the chair if they want to or kind of, do you know what I mean? They don’t need to be worried about that, Oh am I sitting up straight or am I making sure that I’m in the right place and the light is OK and you know all this kind of stuff, I think there’s kind of an ease or something about the phone, maybe?”

(Trainee clinical psychologist, Provider 7)

## Discussion

### Main findings

This study explored the perspectives and experiences of stakeholders of the implementation of remote mental health consultations during the COVID-19 pandemic. The present study employed the Consolidated Framework of Implementation Research (CFIR) to understand the complex factors relevant to implementation at this unique point in time. Participants recognised the *relative advantages* of remote consultations as increasing access to care, convenience, and generally allowing for a more service user-centred model of care. The availability of training and expert guidance and the ease of use of video platforms were also acknowledged as facilitators.

In contrast, providers and managers described the *incompatibility* of remote consultations with existing workflows and increased workload as barriers to implementation. Other barriers included service users’ lack of access to devices and reliable internet connection *(users’ needs and resources)* and changes to the dynamic of the service user-provider relationship (*self-efficacy).* The interplay of various factors was evident in the instances where video consultations were not implemented and replaced with phone consultations. The presence of quickly-changing, and sometimes contradictory, guidelines and regulations was a source of confusion and frustration for providers, occurring alongside the swiftly-changing context of the COVID-19 pandemic. The interviews highlighted the importance of *knowledge and beliefs* to the implementation of remote consultations, particularly surrounding views of the deficits of remote care in terms of fostering a therapeutic environment and relationship. Discourse about the perceived lesser “value” of remote consultations when compared to in–person consultations emphasised these views.

Despite these barriers, participants described making remote consultations work in a way that balanced servicer users’ needs, preferences, and resources with providers’ beliefs. Providers and organisations had to quickly adapt to this new way of providing care, navigating new challenges, learning new skills, and addressing novel issues. This quick transition left little time for planning, and there was a sense of remote consultations not being an optimal solution to a problem, but being better than nothing. In this time of unprecedented stress, fear of the unknown and lack of prior experience with video consultations contributed to these challenges and barriers. With time and experience, some of these challenges were resolved while some remain as likely barriers to future implementation.

### Comparison with previous literature

This study adds to the growing literature on the rapid adoption of telemental health during the COVID-19 pandemic, our findings aligning with this. The convenience and accessibility of telemental health has been widely reported by mental health providers and service users [9, 37]. The present study highlights that this convenience may come at the risk of psychological distance entering the provider-service user relationship. Barriers relating to the *compatibility* construct were reported by Budhwani et al. [7], including changes in workflow and increased provider effort. In the present study, an important distinction was acknowledged between phone and video consultations, whereby providers in general practice described being unsuccessful in implementing video consultations due to incompatibility with workflows and systems. A recent qualitative study by Greenhalgh et al. [38] aimed to shed light on why video consultations are less likely to be implemented in general practice.

Whilst recent studies [7] have reported the impeded therapeutic relationship as a challenge of telemental health, changes to the dynamic of the traditional patient-provider relationship, as discussed in this study, seem to be less present. Perhaps, a reason for these perceived changes is the lack of provider experience with telemedicine in Ireland prior to the pandemic and lack of service user expectations about remote care. In addition, the advantage of remote care of expanding reach to service users has been identified in the literature [9]. However the issue of trying to facilitate increased demand arising from this increased reach has not been fully explored, as many previous studies have been conducted in hospital outpatient settings restricted to serving certain geographical area. This issue is pertinent given the legal and safety considerations of working with service users in different jurisdictions to the provider.

### Implications for practice and research

The findings suggest a number of implications for the future use of remote mental health consultations. In our study, providers described questioning their ability to manage novel, challenging situations, such as managing patients' distress remotely. Providers also discussed feelings of ineffectuality in trying to deal with circumstances outside of their control. Equipping providers with the necessary skills to deal with these new situations through training and expert guidance would be beneficial. As reported in this study, managers highlighted the importance of having access to an expert to provide advice on best-practice. Similarly, the therapeutic relationship appeared as an important consideration in relation to the perceived quality of remote consultations. Creating a safe, contained therapeutic environment seemed to be important to fostering this relationship, but service users did not feel that their home environment was conducive to therapeutic work. Considerations need to be given to ways in which the therapeutic environment can be practically created, such as the physical space in which remote consultations are conducted and encouraging clients to participate from a private, safe space.

In addition to environmental facilitators, service users should be informed of strategies to enhance the creation of the therapeutic relationship, such as using video over phone and having both face and body visible to the provider. Further suggestions are discussed in detail elsewhere [39]. An important consideration relating to the aforementioned point is that service users may be quick to dismiss therapy or care if they experience difficulty with building a connection with their provider. This difficulty may be inadvertently attributed to the provider, type of therapy, or other common factors, rather than considering the online format as a contributor to this challenge. This is an important consideration as some mental health services are entirely online, without the option for in-person contact. One practical suggestion is to have an informal “check-in” between provider and service user, in which they discuss the progress of the service user and their comfort and satisfaction with the online format.

Whilst this study aimed to explore the perspectives of various stakeholders across multiple settings, it may be useful for organisations to conduct formal evaluations of their services to understand context-specific factors relevant to the future use of remote mental health consultations. In particular, an evaluation of users’ needs and resources would be beneficial to organisations given their relative importance to implementation. As noted by Becker et al. [40], the COVID-19 pandemic has highlighted the importance of *outer setting* factors to the implementation of health services and it is now important to consider how knowledge of these factors can be used to increase uptake. For example, acknowledging the digital literacy of service users was an important consideration in this study, which heightens the importance of investigating ways in which digital literacy can be improved.

With this increase of research exploring service users’ and providers’ experiences of telemental health, there is a dearth of literature that has explored telemental health non-adoption during the pandemic. While our study presented challenges of, and barriers to, implementation, the views of those who did not want to participate in remote mental health care are disproportionally excluded in the literature. Further research is needed given the risk of digital exclusion that many service users’ face, particularly if services were to move to fully remote. Furthermore, providers and managers described working with diverse, vulnerable populations, such as international refugees and members of the Irish Traveller Community. Marginalised groups, such as these, may have unique circumstances that may impact their participation in remote mental health care, such as language barriers, use of interpreters, stigma, and low digital literacy. Further research with these populations to understand the cultural barriers to accessing remote mental health care would be beneficial, particularly given the recent migration of Ukrainian refugees across Europe. A final area of potential future research is the environmental benefits of remote mental health care. As discussed in this study, the reduction in travel was a time- and cost- saving benefit for service users, but may also have sustainability benefits that are worth further exploration. This is of particularly relevance to centralised health care, whereby service users are required to travel long distances to access specialist care [41].

### Limitations and strengths

The study findings should be considered in the context of its limitations. As recruitment was primarily conducted online, there may be a bias towards participants who are digitally literate, and the responses may not be representative of all those who partook in remote consultations. The risk of this bias was lessened by offering phone interviews. In addition, the majority of participants worked and lived in urban areas, which disproportionally represent these populations, particularly considering remote care may face additional challenges in rural areas [42]. The single analysis of a small, heterogeneous sample of may be viewed as a potential limitation, however there was high comparability between stakeholders, and similarities and discordances were discussed. Furthermore, within qualitative implementation research, depth of information within a small sample of key stakeholders is considered preferable to superficial information across a larger sample [24]. A final limitation was the use of a single coder to analyse the data.

A strength of this study is the pragmatic and novel use of the CFIR in a qualitative study to explore the rapid implementation of remote mental health consultations in an unplanned and naturalistic context. The most relevant constructs were chosen as broad themes. As mentioned, *users’ needs and resources* was a particularly salient construct that was present in findings described across other themes, such as *relative advantage*. By attempting to use the CFIR to organise our findings, all findings could not fit under this framework, but unique findings were discussed within the context of the framework nevertheless. A further strength was exploring the views of a diverse group of stakeholders, including service users, given the importance of including their voice to understand implementation challenges and successes [12]. The inclusion of providers and managers working with various populations, such as adults, adolescents, and refugees, is a novel strength of this study.

## Conclusions

This study described the perspectives and experiences of service users, mental health providers, and other stakeholders involved in the implementation of remote mental health consultations during the COVID-19 pandemic. The rapid adoption of remote mental health consultations allowed for the continuation of care, but not without considerable changes to service user-provider relationships, processes, and workflows. This study found that whilst remote mental health consultations were appreciated, constant comparison with in-person care lessened the perceived value of remote consultations as an effective form of mental health care delivery. To ensure the appropriate use of remote mental health consultations, considerations need to be given to the creation of a safe, therapeutic space that is conducive to service user engagement. Furthermore, ensuring providers feel competent and embrace the change in dynamic from a traditional in-person consultation appears important to effective adoption.

## Electronic supplementary material

Below is the link to the electronic supplementary material.

Additional file 1: Interview guide for mental health providers

Additional file 2: COREQ Checklist

Additional file 3: Participant characteristics

## Data Availability

The datasets generated and/or analysed during the current study are not publicly available due to the protection of the privacy and confidentiality of the participants but are available from the corresponding author on reasonable request.

## Abbreviations

CFIR: Consolidated Framework for Implementation Research
GP: General practitioner
PPI: Patient and Public Involvement

## References

[1] Batastini AB, Paprzycki P, Jones ACT, MacLean N (2021). Are videoconferenced mental and behavioral health services just as good as in-person? A meta-analysis of a fast-growing practice. Clin Psychol Rev.

[2] Guaiana G, Mastrangelo J, Hendrikx S, Barbui C (2021). A systematic review of the Use of Telepsychiatry in Depression. Community Ment Health J.

[3] Thomas N, McDonald C, de Boer K, Brand RM, Nedeljkovic M, Seabrook L (2021). Review of the current empirical literature on using videoconferencing to deliver individual psychotherapies to adults with mental health problems. Psychol Psychother.

[4] Cowan KE, McKean AJ, Gentry MT, Hilty DM (2019). Barriers to Use of Telepsychiatry: Clinicians as gatekeepers. Mayo Clin Proc.

[5] Scott Kruse C, Karem P, Shifflett K, Vegi L, Ravi K, Brooks M (2018). Evaluating barriers to adopting telemedicine worldwide: a systematic review. J Telemed Telecare.

[6] Nicholas J, Bell IH, Thompson A, Valentine L, Simsir P, Sheppard H (2021). Implementation lessons from the transition to telehealth during COVID-19: a survey of clinicians and young people from youth mental health services. Psychiatry Res.

[7] Budhwani S, Fujioka JK, Chu C, Baranek H, Pus L, Wasserman L (2021). Delivering Mental Health Care virtually during the COVID-19 pandemic: qualitative evaluation of provider experiences in a scaled context. JMIR Form Res.

[8] Gullslett MK, Kristiansen E, Nilsen ER (2021). Therapists’ experience of Video Consultation in Specialized Mental Health Services during the COVID-19 pandemic: qualitative interview study. JMIR Hum Factors.

[9] Al-Mahrouqi T, Al-Alawi K, Al-Alawi M, Al Balushi N, Al Ghailani A, Al Sabti H (2022). A promising future for tele-mental health in Oman: a qualitative exploration of clients and therapists’ experiences. Sage Open Med.

[10] Haxhihamza K, Arsova S, Bajraktarov S, Kalpak G, Stefanovski B, Novotni A (2021). Patient satisfaction with Use of Telemedicine in University Clinic of Psychiatry: Skopje, North Macedonia during COVID-19 pandemic. Telemed J E Health.

[11] Yellowlees P, Nakagawa K, Pakyurek M, Hanson A, Elder J, Kales HC (2020). Rapid Conversion of an Outpatient Psychiatric Clinic to a 100% virtual telepsychiatry clinic in response to COVID-19. Psychiatr Serv.

[12] Guinart D, Marcy P, Hauser M, Dwyer M, Kane JM (2020). Patient attitudes toward Telepsychiatry during the COVID-19 pandemic: a Nationwide, Multisite Survey. JMIR Ment Health.

[13] Li H, Glecia A, Kent-Wilkinson A, Leidl D, Kleib M, Risling T (2022). Transition of Mental Health Service Delivery to Telepsychiatry in response to COVID-19: a Literature Review. Psychiatr Q.

[14] Galvin E, Desselle S, Gavin B, Quigley E, Flear M, Kilbride K (2022). Patient and provider perspectives of the implementation of remote consultations for community-dwelling people with mental health conditions: a systematic mixed studies review. J Psychiatr Res.

[15] Johnson S, Dalton-Locke C, Vera San Juan N, Foye U, Oram S, Papamichail A (2021). Impact on mental health care and on mental health service users of the COVID-19 pandemic: a mixed methods survey of UK mental health care staff. Soc Psychiatry Psychiatr Epidemiol.

[16] Schlief M, Saunders KRK, Appleton R, Barnett P, Vera San Juan N, Foye U (2022). Synthesis of the evidence on what works for whom in Telemental Health: Rapid Realist Review. Interact J Med Res.

[17] Bleyel C, Hoffmann M, Wensing M, Hartmann M, Friederich HC, Haun MW (2020). Patients’ perspective on Mental Health specialist video consultations in primary care: qualitative Preimplementation Study of Anticipated benefits and barriers. J Med Internet Res.

[18] Grol R, Wensing M, Eccles M, Davis D. Improving patient care: the implementation of change in health care. John Wiley & Sons; 2013.

[19] Olwill C, Mc Nally D, Douglas L (2021). Psychiatrist experience of remote consultations by telephone in an outpatient psychiatric department during the COVID-19 pandemic. Ir j psychol Med.

[20] Liberati E, Richards N, Parker J, Willars J, Scott D, Boydell N (2021). Remote care for mental health: qualitative study with service users, carers and staff during the COVID-19 pandemic. BMJ Open.

[21] Haidous M, Tawil M, Naal H, Mahmoud H (2021). A review of evaluation approaches for telemental health programs. Int J Psychiatry Clin Pract.

[22] Moeller AM, Hansen JP, Andersen PT. Patients’ experiences of home-based psychotherapy via videoconference: A qualitative study. Arch Psychiatr Nurs. 2022;39:91–6. 10.1016/j.apnu.2022.03.004 35688550

[23] Uscher-Pines L, Sousa J, Raja P, Mehrotra A, Barnett ML, Huskamp HA (2020). Suddenly becoming a “Virtual Doctor”: experiences of Psychiatrists transitioning to Telemedicine during the COVID-19 pandemic. PS.

[24] Hamilton AB, Finley EP (2019). Qualitative methods in implementation research: an introduction. Psychiatry Res.

[25] Braun V, Clarke V (2006). Using thematic analysis in psychology. Qual Res Psychol.

[26] Damschroder LJ, Aron DC, Keith RE, Kirsh SR, Alexander JA, Lowery JC (2009). Fostering implementation of health services research findings into practice: a consolidated framework for advancing implementation science. Implement Sci.

[27] Kirk MA, Kelley C, Yankey N, Birken SA, Abadie B, Damschroder L (2016). A systematic review of the use of the Consolidated Framework for implementation research. Implement Sci.

[28] Batsis JA, McClure AC, Weintraub AB, Sette D, Rotenberg S, Stevens CJ (2020). Barriers and facilitators in implementing a pilot, pragmatic, telemedicine-delivered healthy lifestyle program for obesity management in a rural, academic obesity clinic. Implement Sci Commun.

[29] Sandelowski M. Whatever happened to qualitative description? Res Nurs Health. 2000;23(4):334–40. 10.1002/1098-240x(200008)23:4<334::aid-nur9>3.0.co;2-g 10940958

[30] Malterud K, Siersma VD, Guassora AD. Sample size in qualitative interview studies: guided by information power. Qual Health Res. 2016;26(13):1753–60 10.1177/1049732315617444 26613970

[31] Sim J, Saunders B, Waterfield J, Kingstone T. Can sample size in qualitative research be determined a priori?. Int J Soc Res Methodol. 2018;21(5):619–34.

[32] Javanparast S, Roeger L, Kwok Y, Reed RL (2021). The experience of australian general practice patients at high risk of poor health outcomes with telehealth during the COVID-19 pandemic: a qualitative study. BMC Fam Pract.

[33] Lincoln YS, Guba EG. Naturalistic inquiry: Sage; 1985.

[34] Nowell LS, Norris JM, White DE, Moules NJ (2017). Thematic analysis: striving to meet the trustworthiness Criteria. Int J Qual Meth.

[35] Tong A, Sainsbury P, Craig J (2007). Consolidated criteria for reporting qualitative research (COREQ): a 32-item checklist for interviews and focus groups. Int J Qual Health Care.

[36] Shenton AK (2004). Strategies for ensuring trustworthiness in qualitative research projects. Educ Inform.

[37] McQueen M, Strauss P, Lin A, Freeman J, Hill N, Finlay-Jones A (2022). Mind the distance: experiences of non-face-to-face child and youth mental health services during COVID-19 social distancing restrictions in western Australia. Aust Psychol.

[38] Greenhalgh T, Ladds E, Hughes G, Moore L, Wherton J, Shaw SE et al. Why do GPs rarely do video consultations? Qualitative study in UK general practice. Br J Gen Pract. 2022;72(718):e351–60. 10.3399/BJGP.2021.0658 PMC8936181 35256385

[39] Simpson S, Richardson L, Pietrabissa G, Castelnuovo G, Reid C. Videotherapy and therapeutic alliance in the age of COVID-19. Clin Psychol Psychother. 2021;28(2):409–21. 10.1002/cpp.2521 PMC7675483 33037682

[40] Becker SJ, Garner BR, Hartzler BJ (2021). Is necessity also the mother of implementation? COVID-19 and the implementation of evidence-based treatments for opioid use disorders. J Subst Abuse Treat.

[41] Mungall IJ (2005). Trend towards centralisation of hospital services, and its effect on access to care for rural and remote communities in the UK. Rural Remote Health.

[42] Berryhill MB, Halli-Tierney A, Culmer N, Williams N, Betancourt A, King M (2019). Videoconferencing psychological therapy and anxiety: a systematic review. Fam Pract.

