# Supplementary material for: Stakeholder perspectives and experiences of the implementation of remote mental health consultations during the COVID-19 pandemic: a qualitative study
Source: BMC Health Serv Res. 2023 Jun 13;23:623. doi: 10.1186/s12913-023-09529-x (PMC10262124; doi:10.1186/s12913-023-09529-x)
Supplement: Supplementary file 1 — Additional file 1: Interview guide for mental health providers [file 12913_2023_9529_MOESM1_ESM.docx]

**Additional file 3**

**Participant characteristics**

Table 1. Mental health provider characteristics

| **Mental health provider** | **Job title** | **Gender** | **Years of practice** | **Previous experience with remote consultations** | **Setting** |
| --- | --- | --- | --- | --- | --- |
| 1 | Psychotherapist | Female | 18 | No | Private |
| 2 | Pharmacist | Female | 11 | No | Hospital outpatient |
| 3 | Clinical psychologist | Female | 8 | No | Community |
| 4 | Psychiatrist | Male | 35 | Yes (Phone) | Outpatient |
| 5 | General practitioner | Male | 35 | Yes (Phone) | General practice |
| 6 | General practitioner | Female | 12 | Yes (Phone) | General practice |
| 7 | Trainee clinical psychologist | Female | Trainee | No | General practice |
| 8 | Trainee clinical psychologist | Female | Trainee | No | General practice |
| 9 | Child and adolescent psychiatrist | Female | 14 | No | Private |

Table 2. Service user characteristics

| **Service user** | **Gender** | **Previous experience with remote consultations** |
| --- | --- | --- |
| 1 | Female | No |
| 2 | Female | No |
| 3 | Male | No |
| 4 | Female | No |
| 5 | Female | No |

Table 3. Manager characteristics

| **Manager** | **Gender** | **Previous experience with remote consultations** | **Setting** |
| --- | --- | --- | --- |
| 1 | Male | Yes (Video) | Counselling organisation |
| 2 | Male | No | Counselling organisation |
| 3 | Female | No | Counselling organisation |
| 4 | Female | N/A | Professional body |
| 5 | Male | No | Counselling organisation |
