# Supplementary material for: Stakeholder perspectives and experiences of the implementation of remote mental health consultations during the COVID-19 pandemic: a qualitative study
Source: BMC Health Serv Res. 2023 Jun 13;23:623. doi: 10.1186/s12913-023-09529-x (PMC10262124; doi:10.1186/s12913-023-09529-x)
Supplement: Supplementary file 3 — Additional file 3: Participant characteristics [file 12913_2023_9529_MOESM3_ESM.docx]

**Additional file 1**

**Interview guide for mental health providers**

**Introduction & audio consent**

Hello [participant name], thank you for agreeing to participate in an interview.

*Describe study and what to expect from interview*.

Do you have any questions before I begin recording?

*Switch audio recorder on*

For the audio recording, can I check that:

- You have read and understood the information sheet?
- You know that your participation in this interview is voluntary and that you can stop the interview at any time?
- You know that quotations from the interview may be used in the findings, but it will not be possible to trace who said them?
- You agree to the interview being audio recorded?
- You consent to take part in the study?

**Introductory questions**

I’m just going to begin with a few introductory questions about your role and familiarity with remote consultations

- So first of all, so I have some basic demographic information, could you please tell me what setting you work in, how many years you have been in practice for, and your gender?
- As I have mentioned, we are interested in remote consultations in mental health care. What is your level of familiarity with using these?
  - Are there any other names you would use for these type of consultations?
- So in the past month how many remote consultations have you undertaken roughly?
  - How does this compare to the number of in-person consultations undertaken?
  - And how would the number of remote consultations compare with in-person consultations compared to during the start of the pandemic.
  - And were these phone or video or both?
- Would you have used remote consultations before the pandemic?
  - What forms? In what cases?

**Adapting to remote consultations**

For my next few questions, I want you to think back to the start of pandemic when remote consultations first came in

- So how did you feel about the move to remote consultations?
- What kinds of workflow or organisational changes were needed to accommodate the move to remote consultations?
- Were there changes in your workload with the move to remote consultations?
- What was the general level of receptivity in your department/organization to adopting remote consultations?
  - Why do you think this was? Do you think it has changed?
- And at this time, how confident did you feel about conducting remote consultations?
  - Why?
  - How has this changed?
- So at the start, what did you find most difficult about undertaking remote consultations?
  - Why?
  - How has this changed?
- Were you aware of any policy, regulations, guidelines that may have impacted the adoption of remote consultations?
  - If yes, in what way would they influence the adoption of remote consultations?
- What level of involvement/support had leadership at your organization had so far with implementing remote consultations?

**Platform**

- Can you talk me through the process of conducting a remote consultation with a service user?
- What platform do you use? What do you think of it?
  - Quality? Complexity? GDPR-compliant?
  - Pros and cons of different platforms?
- Did you receive any resources and information for implementing remote consultations?
  - IT Support? Training?
  - If yes, what did you think of them?
  - If no, what would you have like to have received?
- Do you have any concerns relating to privacy of these platforms?
  - GDPR? Platforms?

**Service users**

So my next few questions relate to your view on service users’ experiences of remote consultations?

- How receptive have service users been to remote consultations?
  - Any changes over time?
  - Any concerns expressed?
  - Any reluctance to participating?
- How well do you think remote consultations meets the needs of service users?
  - So for example, improving outcomes or their satisfaction?
- What is your impression of service users’ experiences with remote consultations?
  - Anecdotal stories of success?

**Comparison with in-person consultations**

- Compared to in-person consultations, what do you think are the advantages of remote consultations?
  - And disadvantages?
- Is there anything that you are unable to do during remote consultations that you would normally do during an in-person visit?
- If you have done consultations by both phone and video, how do they differ?

**Current situation & recommendations**

My final few questions are about the present use of remote consultations and the future of remote consultations in mental health care.

- Do you think there is currently a strong need for remote consultations?
  - Why?/Why not?
- Are remote consultations a priority in your organization/hospital at the moment?
- What changes would you make to remote consultations to make them fit or work more effectively in your setting?
  - What aspects would you have liked to change, but couldn’t?
  - What barriers exist to making these changes?
- Was there anything that worked particularly well in relation to the implementation of remote consultations?
- How best should we use remote consultations in mental health care going forward?
  - Which type of service user may benefit most?
  - What type of consultations work best over phone or video?
- Based on your experiences, would you recommend continuing remote consultations at your practice?
  - Why?/Why not?
- Is there anything else you would like to add about your experience with remote consultations?

**Closing**

Ok, so that’s all my questions.

Thank you very much for your time. Do you have any other questions for me?
